# Treatment patterns in psoriatic arthritis patients newly initiated on oral nonbiologic or biologic disease-modifying antirheumatic drugs

**DOI:** 10.1186/s13075-014-0420-5

**Published:** 2014-08-22

**Authors:** Huabin F Zhang, Geneviève Gauthier, Robert Hiscock, Jeffrey R Curtis

**Affiliations:** Celgene Corporation, 86 Morris Ave, Summit, NJ 07901 USA; Analysis Group Inc, 111 Huntington Ave #10, Boston, MA 02199 USA; University of Alabama at Birmingham, 510 20th St. South FOT 802D, Birmingham, AL 35294 USA

## Abstract

**Introduction:**

This study aimed to describe treatment changes (discontinuation, switching, and therapy add-on) following the initiation of biologic or nonbiologic oral disease-modifying antirheumatic drugs (DMARDs) in psoriatic arthritis (PsA) patients.

**Methods:**

Adult patients with ≥2 PsA diagnoses from physician office visits, initiated on a biologic or nonbiologic oral DMARD, were selected from the Truven Health Analytics MarketScan® Research Database (2005 to 2009). Patients were required to have continuous insurance coverage ≥6 months prior to and ≥12 months post index date (first prescription fill date). Treatment discontinuation, treatment switch, and therapy add-on were captured over the 1 year period following the index date. Treatment changes were described separately for patients initiated on nonbiologic and biologic DMARDs.

**Results:**

A total of 1,698 and 3,263 patients were initiated on an oral nonbiologic DMARD and biologic DMARD respectively. For patients initiated on nonbiologic DMARDs, 69% had ≥1 therapy change over the 12 month study period (median time 85 days). Among patients who had a therapy change, 83% discontinued, 29% switched therapy (64% switched to a biologic DMARD), and 25% had a therapy add-on (76% added-on with a biologic DMARD). For patients initiated on a biologic DMARD, 46% had ≥1 therapy change (median time 110 days). Among patients who had a therapy change, 100% discontinued, 25% switched therapy (92% switched to another biologic DMARD), and 7% had a therapy add-on with a nonbiologic DMARD.

**Conclusion:**

This study suggests that PsA patients newly initiated on a nonbiologic/biologic DMARD do not remain on the index treatment for a long period of time. A better understanding of factors related to these early treatment changes in PsA patients is needed.

## Introduction

Psoriatic arthritis (PsA), an idiopathic, chronic, and often progressive immune-mediated autoinflammatory arthritis, affects peripheral and axial joints, nails and entheses, and is typically accompanied by psoriatic skin lesions [1,2]. Although phenotypically heterogeneous, PsA symptoms typically include joint pain, stiffness, swelling, nail psoriasis, dactylitis and generalized fatigue [1,3]. In the United States, the prevalence of PsA is estimated to be between 0.10% and 0.25% of the overall population [1].

The immediate treatment goals in PsA include mitigating joint pain and swelling, skin lesions, disease progression, and systemic sequelae [3,4]. The ultimate treatment goal - disease remission - is characterized by the absence of clinically discernible disease activity and the potential for joint healing [4,5]. Pharmacotherapy for PsA encompasses nonsteroidal anti-inflammatory drugs (NSAIDs) and intra-articular corticosteroid injections to manage musculoskeletal pain, stiffness, and swelling, as well as nonbiologic and biologic disease-modifying antirheumatic drugs (DMARDs), which have the potential to attenuate joint damage and promote disease remission [6].

American Academy of Dermatology (AAD) and the European League against Rheumatism (EULAR) recommended treatment guidelines for PsA advocate a stepwise approach to treatment based on symptom severity, joint involvement, and the extent of inflammation [6-8]. Mild PsA is typically treated with NSAIDs or intra-articular corticosteroid injections [6,9]. If a satisfactory response is not achieved after 3 months, or if there is evidence of persistent inflammation or of erosive or polyarticular disease, the guidelines suggest the option of using a traditional oral nonbiologic DMARD, such as methotrexate (MTX) [7-9]. Yet the clinical data supporting the use of MTX as a disease-modifying agent in PsA remain limited [10-13]. In a recent double-blind, randomized, placebo-controlled study in patients with active PsA, MTX treatment failed to improve objective assessments of synovitis or of tender and swollen joint counts, despite improvements in skin scores and patients’ and assessors’ global evaluations [13]. If traditional oral nonbiologic DMARDs cannot properly control the signs and symptoms of PsA, the guidelines suggest the use of biologics such as etanercept and adalimumab [7]. Biologics have demonstrated robust efficacy in PsA to date [2]. A recent study investigating potential effects of MTX co-medication with TNF inhibitor (TNFi) - a type of biologic - on treatment response and persistence in PsA patients found a benefit in terms of treatment persistence of using MTX concomitantly with another TNFi [14]. Despite the treatment guidelines and clinical studies that demonstrate the benefits and risks of oral DMARDs and biologics in PsA, it remains unclear how physicians generally approach the management of PsA. The main objective of this study was to describe treatment patterns (treatment discontinuation, treatment switches, and therapy add-ons) for PsA patients newly treated with oral nonbiologic or biologic DMARDs in a real-world setting.

## Methods

### Data source

This retrospective study used data from the US-based Truven Health Analytics MarketScan® Research Databases, acquired between the first quarter (Q1) of 2005 and Q4 of 2009. The database included approximately 25 million individuals, annually covered by 130 health plans and self-insured employers. The database covers all census regions in the US and contains information on patient demographics, enrollment history, claims for inpatient and outpatient medical services, and pharmacy claims. Patient data were de-identified and comply with the patient confidentiality requirements of the Health Insurance Portability and Accountability Act. Therefore, Institutional Review Board approval was not required.

### Sample selection and construction

Adult PsA patients newly treated with oral nonbiologic DMARDs or biologic DMARDs were selected in this study. Patients initiated on nonbiologic DMARDs were required to be naïve to any biologic or nonbiologic DMARDs recommended for PsA before the first oral nonbiologic DMARD initiation date (index date). Biologic DMARD patients may have been pre-treated with a nonbiologic DMARD before the index date. Adult patients with active PsA were defined as those who were at least 18 years of age at the index date and had at least two physician PsA diagnoses (ICD-9-CM code: 696.0x) from two separate office visits over the 18-month observation period (that is, at least one diagnosis during the 6-month period before the index date (baseline period), and at least one during the 12-month period after the index date (study period)). Patients who did not have at least a 6-month baseline period or 12-month study period in the database were excluded from the study.

Oral nonbiologic DMARDs considered included MTX, cyclosporine, leflunomide, mycophenolate, gold compounds, antimalarials, minocycline, penicillamine, azathioprine, and sulfasalazine. Due to the wide use of MTX in PsA, a subgroup analysis was conducted for patients newly initiated on MTX. Biologic DMARDs in the study included etanercept, infliximab, golimumab, certolizumab pegol, adalimumab, anakinra, abatacept, and rituximab based on clinical use although some do not have indication in PsA. Patients were excluded if they had a diagnosis of ankylosing spondylitis (AS) (ICD-9-CM code: 720.0x) at any time before the index date.

Patients newly initiated on oral nonbiologic DMARDs and biologic DMARDs were analyzed separately during the baseline and study period. These two samples were not mutually exclusive. For example, a patient initiating therapy on an oral nonbiologic DMARD who later switched to a biologic DMARD could be included in both samples if the two initiation dates met all the aforementioned selection criteria. However, this had no impact on the study analyses and interpretation of the results, given that the two samples were analyzed independently, and no direct statistical analyses were performed between the two samples.

### Outcome measures

#### Therapy change

As defined subsequently, therapy change was defined as the discontinuation of the index treatment (that is, the nonbiologic or biologic DMARD initiated at the index date), a switch from the index treatment to another treatment, or a therapy add-on. Patients may have had more than one therapy change over the study period.

#### Discontinuation

Treatment discontinuation was defined as the first occurrence of a treatment interruption of at least 60 consecutive days between the end of the drug supply for a prescription of the index treatment and the beginning of a next prescription for the index treatment, or the end of the one-year study period, whichever occurred first.

#### Treatment switch

A treatment switch was defined as the initiation of a treatment other than the index DMARD within 60 days of the interruption of the index treatment. For patients initiated on an oral nonbiologic or on a biologic DMARD, other nonbiologic (either oral or subcutaneous), or biologic DMARDs were considered as switch treatments. Additionally, as patients initiated on a biologic could have used nonbiologic DMARDs prior to the index date, the nonbiologic treatment (either oral or subcutaneous) to which the patient was being switched could not have been taken during the 60-day period prior to the switch date. For patients who initiated concomitant treatments on the index date, any treatments used at the index date were not considered potential switch treatments. Treatment switches were captured from the index date up to 60 days after the first discontinuation of the index treatment, or up to the end of the study period, whichever occurred first. All treatments initiated within the 60 days following treatment discontinuation were reported as a switch.

#### Therapy add-on

Therapy add-on was defined as the use of a DMARD treatment other than the treatment used at the index date for at least 28 consecutive days before the discontinuation of the index therapy. For patients initiated on an oral nonbiologic DMARD, the use of any other nonbiologic DMARDs (either oral or subcutaneous), or biologic DMARDs were considered potential therapy add-on treatments. For patients initiated on a biologic DMARD, nonbiologic DMARDs (either oral or subcutaneous) were considered potential therapy add-on treatment agents. Additionally, as patients who were initiated on a biologic DMARD may have used nonbiologic DMARDs prior to the index date, the DMARD used for add-on was required to have not been taken during the 60-day period prior to the start of therapy add-on. For patients who initiated concomitant treatments at the index date, any treatments used at the index date were not considered potential add-on treatments. Add-on rates were captured over the period spanning from the index date up to the discontinuation of the index treatment, or until the end of the study period, whichever occurred first.

### Sensitivity analyses

It may be difficult in claims data to ensure that the selected PsA patients have been accurately diagnosed for PsA, particularly since some of the core symptoms of PsA are also present in AS or rheumatoid arthritis (RA). However, AS and RA may similarly lead to patient misclassification as a PsA diagnosis, or may have overlapping features with PsA. As a result, two sensitivity analyses were performed to test the robustness of our findings; (1) as AS patients were excluded in the main analysis, the first sensitivity analysis was conducted without excluding patients with AS, and (2) as RA patients were included in the main analysis, the second sensitivity analysis excluded patients diagnosed with RA.

Additionally, as a supplemental sensitivity analysis, discontinuation was defined as the first occurrence of a treatment interruption of at least 90 consecutive days between the end of the drug supply (for a prescription of the index treatment) and the beginning of a next prescription (for the index treatment), or the end of the one-year study period, whichever occurred first.

### Statistical analyses

Baseline data were analyzed descriptively and reported separately for patients initiated on an oral nonbiologic DMARD, for patients initiated on a biologic DMARD, and for the subgroup of patients initiated on MTX. Reported characteristics included demographics (age, gender), treatment characteristics, specialist encounters, and comorbidities. The Charlson comorbidity index (CCI) adapted by Deyo for administrative databases was used to assess the level of comorbidity associated with each PsA cohort [15].

Treatment patterns were reported separately for patients initiated on an oral nonbiologic DMARD, on a biologic DMARD, and for patients from the MTX subgroup. The proportions of patients who discontinued the index treatment, switched to another DMARD, had a therapy add-on, or had any therapy changes over the 12-month study period were reported. Additionally, the proportion of patients who remained on the index treatment, and the proportion of patients using any nonbiologic or biologic DMARD at the end of each month during the 12-month study period were reported. No comparative analyses were performed between samples. As patients could have more than one type of treatment change, the percentage of all changes does not always add up to 100%.

The median time to the observed therapy change was also reported. Median time to therapy change was calculated among patients who incurred the studied therapy change as the number of days between the index date and the date of the studied therapy change.

## Results

**Figure 1:**
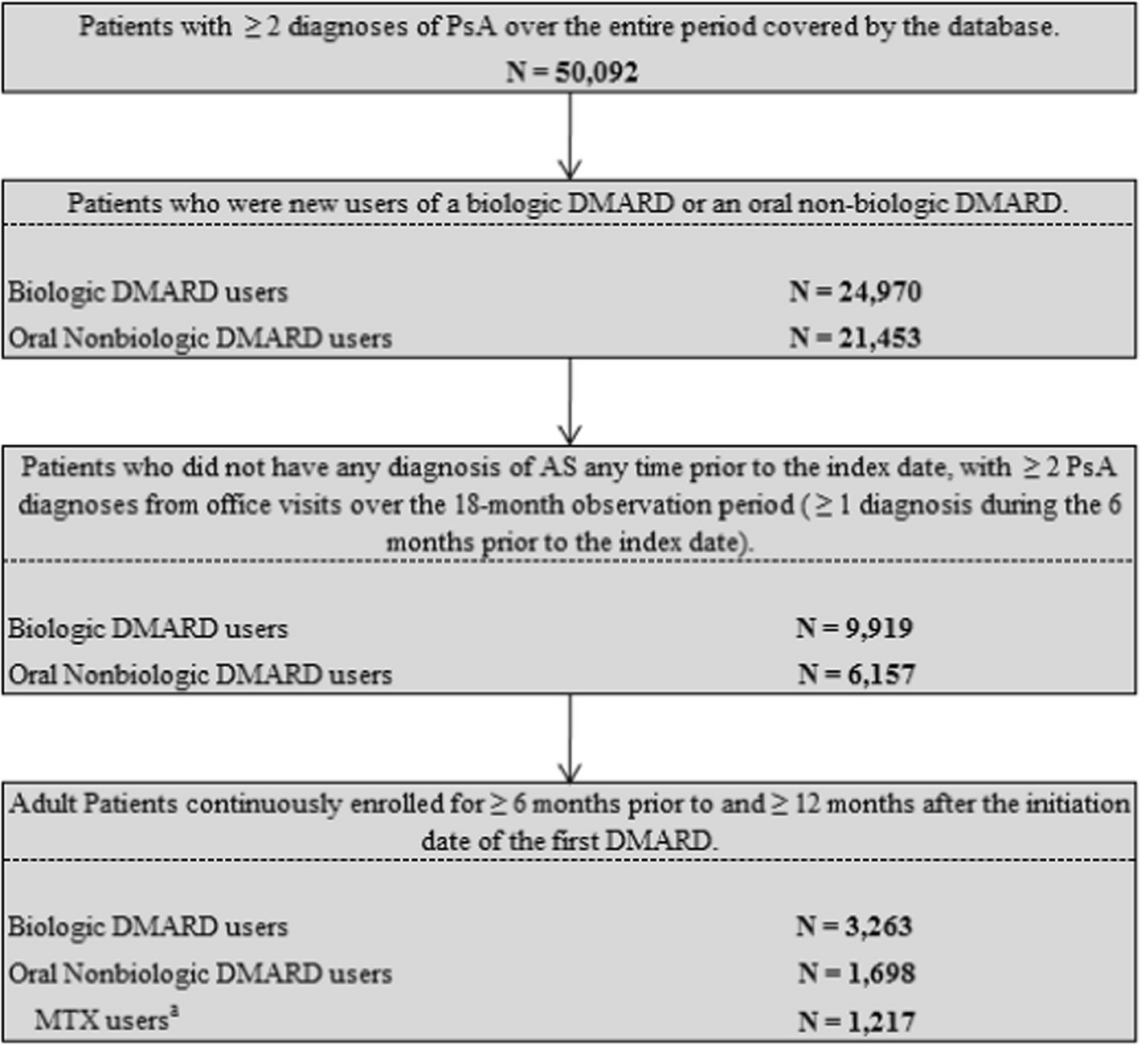
**Sample selection criteria results.** ^a^MTX users are a subgroup of the oral nonbiologic DMARD users. DMARD, disease-modifying antirheumatic drug; MTX, methotrexate; NSAID, nonsteroidal anti-inflammatory drug; PsA, psoriatic arthritis.

Overall, 1,698 patients met the sample selection criteria and initiated an oral nonbiologic DMARD on the index date, with the majority being initiated on MTX (71%, n = 1,217). Additionally, 3,263 patients met the sample selection criteria and initiated a biologic DMARD treatment on the index date (Figure 1).

### Baseline characteristics

Patients’ average age was approximately 50 years, and a slight majority (54%) was female (Table 1). Over 60% of patients were seen by a rheumatologist during the baseline period or at the index date, and approximately a quarter were seen by a dermatologist. Average CCI was 0.47, 0.44, and 0.52 for patients initiated on an oral nonbiologic DMARD, MTX, and biologic DMARD therapy, respectively. The most common comorbidities during the baseline period were psoriasis (approximately 35%), hypertension (approximately 20%), diabetes (approximately 11%), and hypothyroidism (approximately 6%) (Table 1).

**Table 1:** **Baseline** ^**a**^ **characteristics**

**Characteristics**	**Oral nonbiologic DMARD patients**	**MTX patients**	**Biologic DMARD patients**
	**N = 1,698**	**N = 1,217**	**N = 3,263**
Demographics			
Age, mean (SD)	49.8 (12.5)	49.8 (12.3)	49.4 (11.6)
Female, number (%)	927 (54.6)	657 (54.0)	1,736 (53.2)
Combination of treatment with another nonbiologic DMARD and/or oral corticosteriod at the index date	168 (9.9)	138 (11.3)	1,512 (46.3)
During the 6-month pre-index period, including the index date			
Specialist encounter, number (%)			
Rheumatologist or Dermatologist	1,175 (69.2)	841 (12.3)	2,347 (71.9)
Rheumatologist	1,055 (62.1)	755 (62.0)	2,008 (61.5)
Dermatologist	384 (22.6)	271 (22.3)	896 (27.5)
During the 6-month pre-index period			
Comorbidities^b,c^, number (%)			
Hypertension	364 (21.4)	260 (21.4)	670 (20.5)
Diabetes	175 (10.3)	129 (10.6)	376 (11.5)
Hypothyroidism	114 (6.7)	81 (6.7)	200 (6.1)
Chronic pulmonary disease	111 (6.5)	72 (5.9)	176 (5.4)
Deficiency anemia	77 (4.5)	62 (5.1)	164 (5.0)
Psoriasis	545 (32.1)	420 (34.5)	1,194 (36.6)
Charlson comorbidity index, mean (SD)	0.47 (0.87)	0.44 (0.84)	0.52 (0.88)
Prior use of NSAID, oral corticosteriod, and nonbiologic DMARDs, number (%)			
NSAIDs	991 (58.4)	701 (57.6)	1,665 (51.0)
Oral corticosteriods	550 (32.4)	396 (32.5)	1,036 (31.7)
Nonbiologic DMARDs	0 (0.0)	0 (0.0)	1,938 (59.4)

^a^The baseline period consisted of the 6-month period prior to the index date. ^b^Elixhauser A, Steiner C, Kruzikas D. Comorbidity Software. January 2004. HCUP Methods Series Report #2004-1. Online, February 6, 2004. US Agency for Healthcare Research and Quality: pp 12-15 [16]. ^c^ Only mental and physical comorbidities where at least one of the reported cohorts had a prevalence of ≥5% were reported in the table. DMARD, disease-modifying antirheumatic drugs; MTX, methotrexate; number, number of patients; NSAID, nonsteroidal anti-inflammatory drug.

During the baseline period, 58% of patients initiated on an oral nonbiologic DMARD were treated with an NSAID, and 32% received oral corticosteroids. For patients initiated on a biologic DMARD, 59% used a nonbiologic DMARD during the baseline period, 51% an NSAID, and 32% an oral corticosteroid. Approximately 10% of patients initiated on an oral nonbiologic DMARD used an oral corticosteroid at the index date, while approximately 46% of patients initiated on a biologic DMARD used a nonbiologic DMARD and/or an oral corticosteroid at the index date (35% used a biologic DMARD and a nonbiologic DMARD, 5% used a biologic DMARD and an oral corticosteroid, and 6% used a biologic DMARD, a nonbiologic DMARD, and an oral corticosteroid). These numbers included patients who initiated a nonbiologic DMARD and/or an oral corticosteroid at the index date or prior to the index date and whose prescription overlapped with the index date. For patients initiated on an oral nonbiologic DMARD, the average total healthcare cost per patient over the 6-month baseline period was $5,377, while the same period yielded an average cost of only $2,476 for patients initiated on a biologic DMARD.

### Therapy changes

#### Oral nonbiologic DMARD users

Overall, 69% of patients initiated on an oral nonbiologic DMARD and 65% of patients in the MTX subgroup had at least one therapy change during the course of the 12-month study. The median time to the first therapy change was 85 days for patients initiated on an oral nonbiologic DMARD and 93 days for the MTX subgroup.

**Figure 2:**
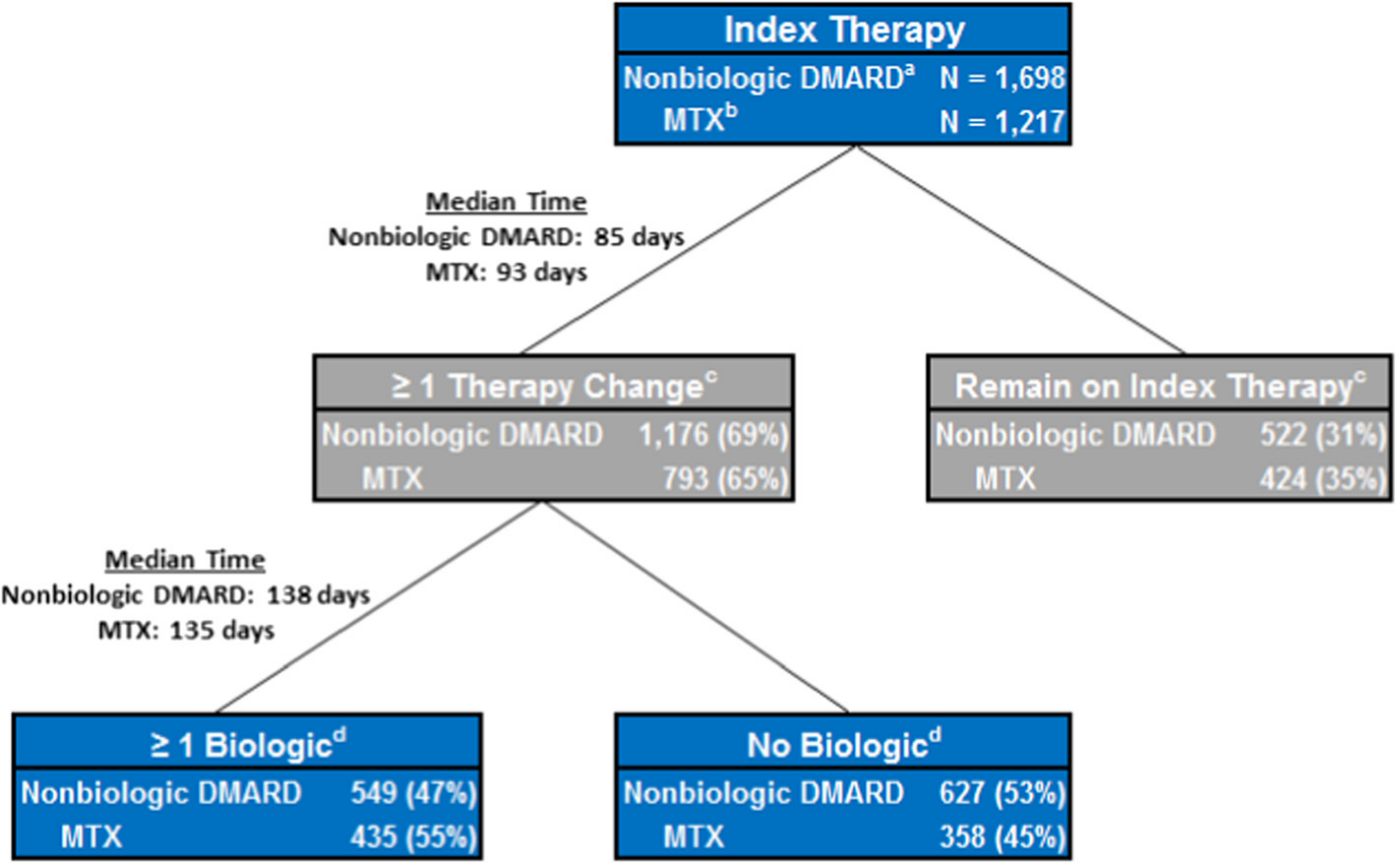
**Biologic use in oral nonbiologic DMARD patients and in MTX patients.** ^a^This refers to oral nonbiologic DMARD users. ^b^MTX users are a subgroup of the oral nonbiologic DMARD users. ^c^These categories presented are mutually exclusive. ^d^These categories presented are mutually exclusive. DMARD, disease-modifying antirheumatic drug; MTX, methotrexate.

Among patients who had at least one therapy change, 47% of the oral nonbiologic DMARD users and 55% of the patients in the MTX subgroup used a biologic DMARD at some point during the 12-month study period. The median time to the first biologic DMARD use was 138 days for oral nonbiologic DMARD users and 135 days for patients in the MTX subgroup (Figure 2).

**Figure 3:**
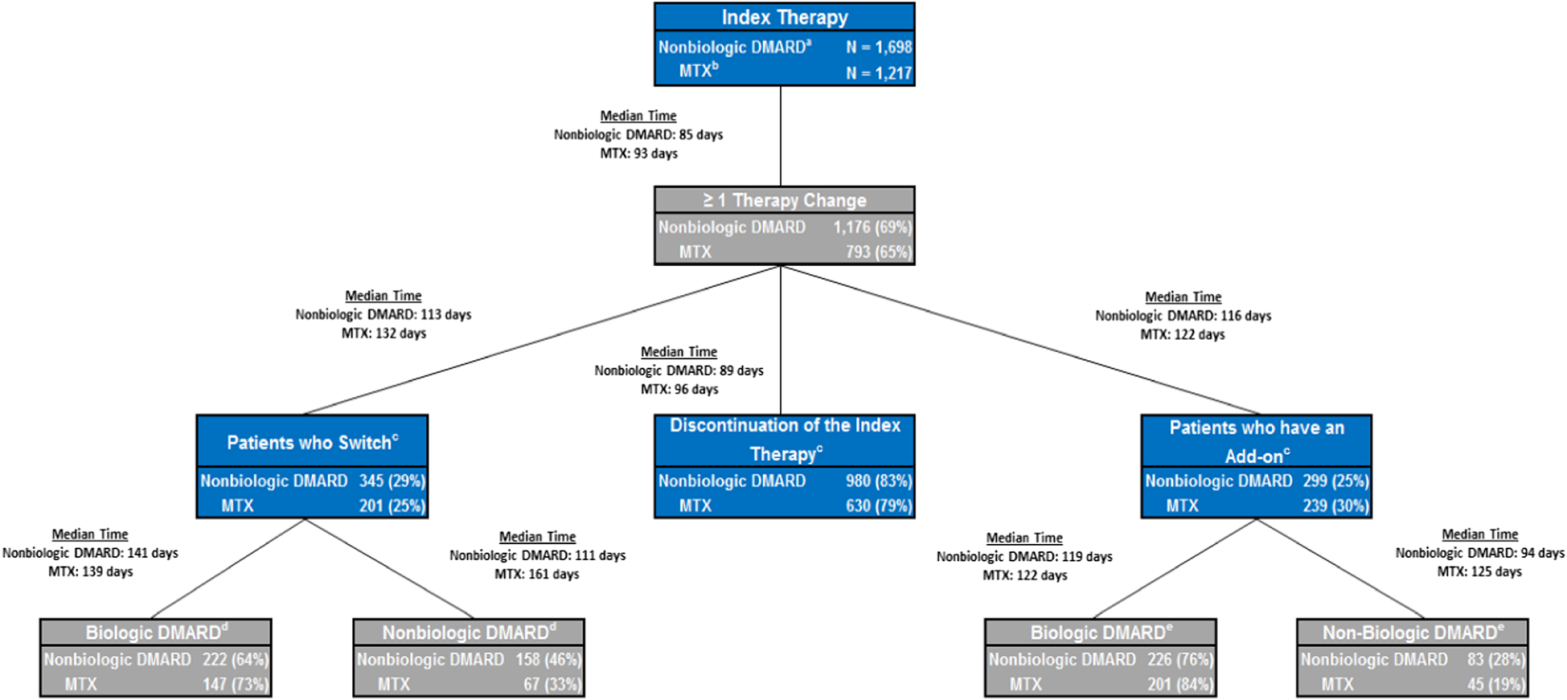
**Detailed schematic of therapy changes in oral nonbiologic DMARD patients and in MTX.** ^a^This refers to oral nonbiologic DMARD users. ^b^MTX users are a subgroup of the oral nonbiologic DMARD users. ^c^These categories presented are not mutually exclusive. ^d^These categories presented are not mutually exclusive. ^e^These categories presented are not mutually exclusive. DMARD, disease-modifying antirheumatic drug; MTX, methotrexate.

Among patients who had at least one therapy change, 83% discontinued treatment (median time: 89 days), 29% switched (median time: 113 days), and 25% had a therapy add-on (median time: 116 days) (Figure 3). Among patients who switched therapies, 64% switched to a biologic DMARD (median time: 141 days), and 46% switched to another nonbiologic DMARD (median time: 111 days). Among patients who had a therapy add-on, 76% of patients added-on with a biologic DMARD (median time: 119 days), and 28% added-on with another nonbiologic DMARD (median time: 94 days).

Among the patients in the MTX subgroup who had at least one therapy change, 79% discontinued (median time: 96 days), 30% had a therapy add-on (median time: 122 days), and 25% switched (median time: 132 days) (Figure 3). Among the patients who switched therapies, 73% switched to a biologic DMARD (median time: 139 days), and 33% switched to another nonbiologic DMARD (median time: 161 days). Among the patients who had a therapy add-on, 84% had a biologic DMARD added (median time: 122 days), and 19% had another non-biologic DMARD added (median time: 125 days).

**Figure 4:**
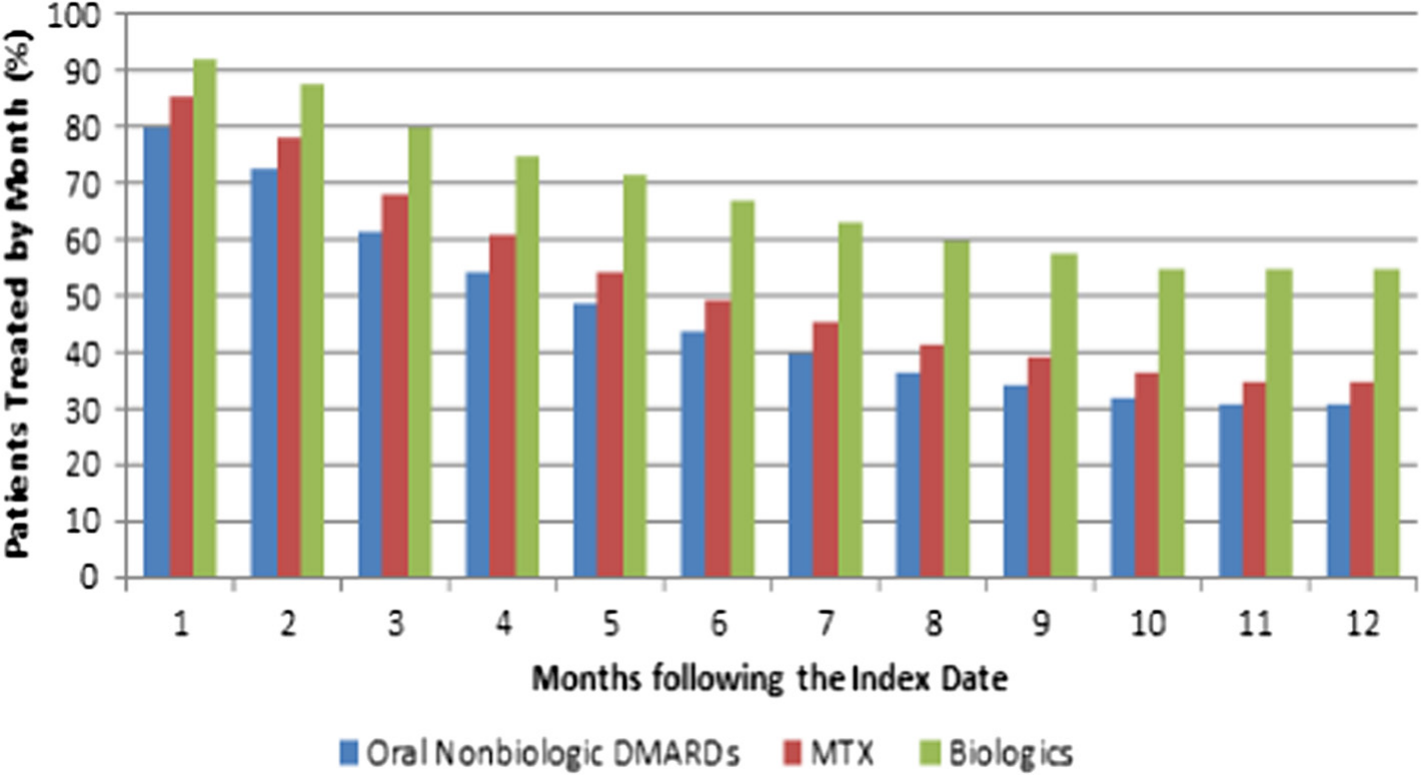
**Patients remaining on the index therapy by month.** DMARD, disease-modifying antirheumatic drug; MTX, methotrexate.

**Figure 5:**
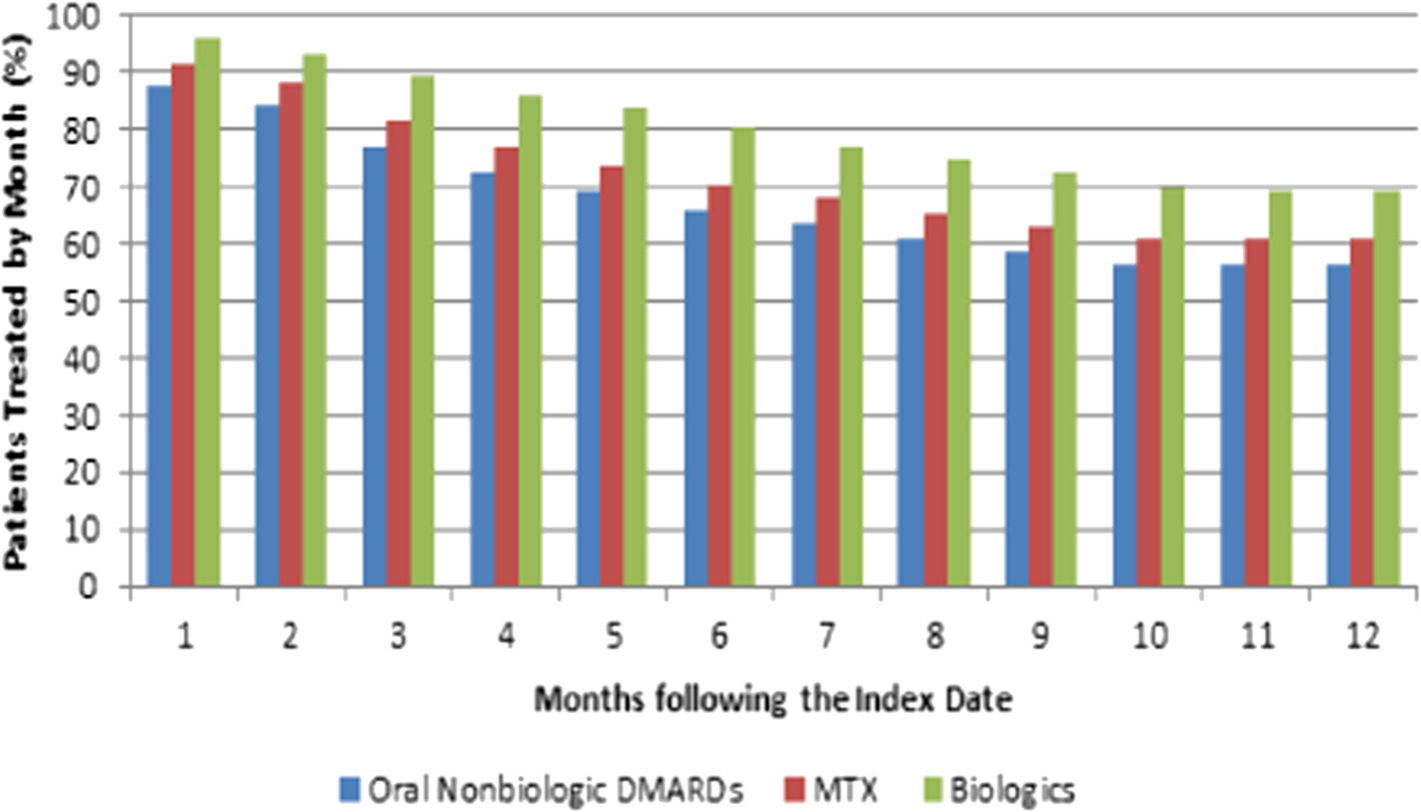
**Patients remaining on any PsA-related therapy by month.** In this figure, only nonbiologic DMARDs and biologic DMARDs are considered as any PsA-related therapy. DMARD, disease-modifying antirheumatic drug; MTX, methotrexate; PsA, psoriatic arthritis.

Among patients initiated on an oral nonbiologic DMARD, 80%, 62%, 43%, and 31% of the patients were still continuously treated with the index therapy by the end of the first, third, sixth, and twelfth month following the index date, respectively (Figure 4). Further, 88%, 77%, 66%, and 56% were still continuously treated with any nonbiologic or biologic DMARD at the end of the first, third, sixth, and twelfth month following the index date, respectively (Figure 5).

#### Biologic DMARD users

**Figure 6:**
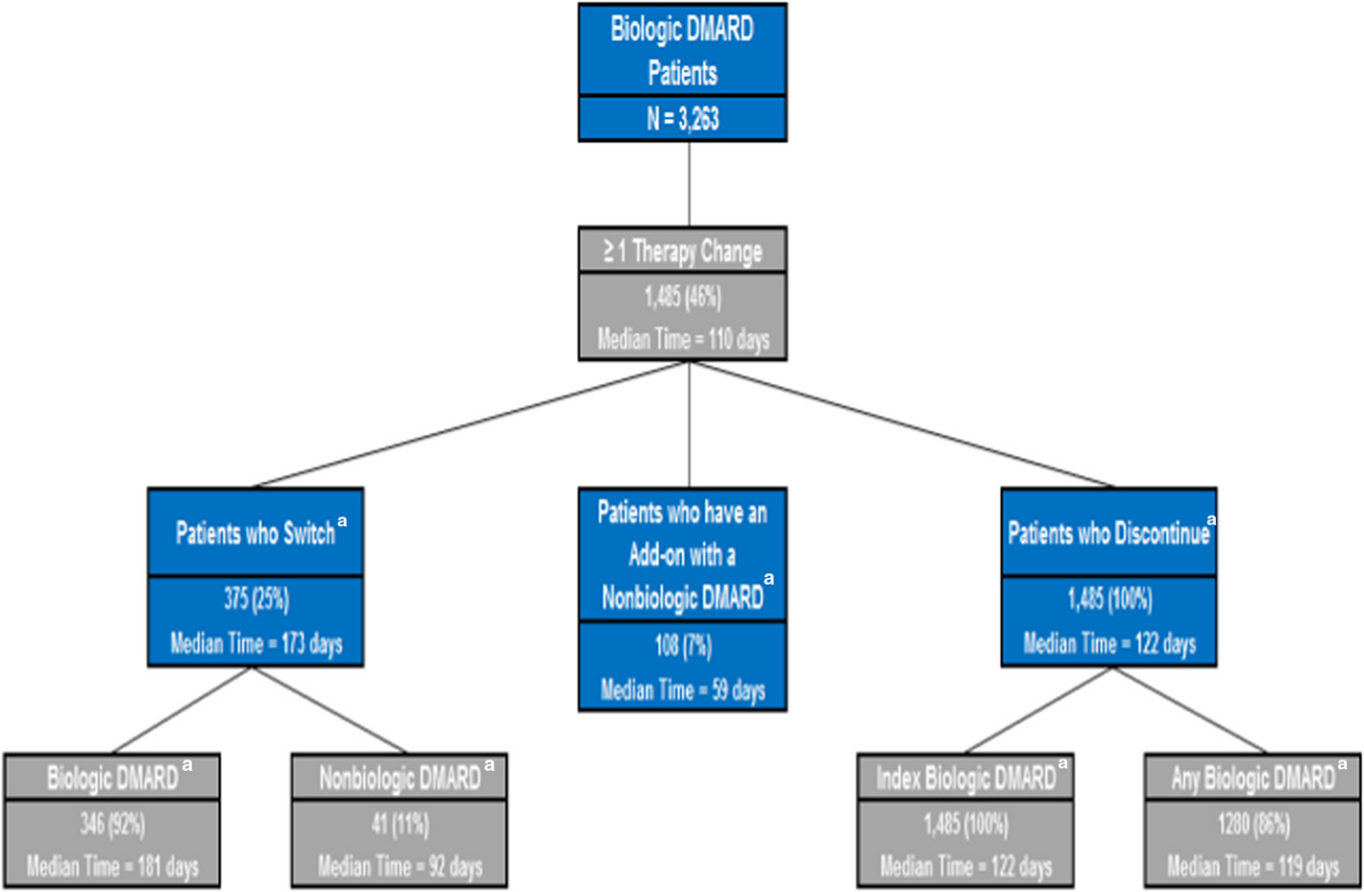
**Detailed schematic of therapy changes in biologic DMARD patients.** ^a^These categories presented are not mutually exclusive.

Overall, 46% of the patients had at least one therapy change during the course of the 12-month study period. The median time to the first therapy change was 110 days. All patients who had at least one therapy change discontinued the index biologic treatment at some point during the 12-month study period (median time: 122 days). Additionally, among patients who had at least one therapy change, 25% of patients switched (median time: 173 days), and 7% of patients had a therapy add-on with a nonbiologic DMARD (median time: 59 days). Among patients who switched therapies, 92% of the patients switched to another biologic DMARD (median time: 181 days), and 11% switched to a nonbiologic DMARD (median time: 92 days) (Figure 6). Moreover, 92%, 80%, 67%, and 54% of patients were still continuously treated with the index therapy by the end of the first, third, sixth, and twelfth month following the index date, respectively (Figure 4), and approximately 96%, 89%, 80%, and 69% were still continuously treated with a nonbiologic or biologic DMARD at the end of the first, third, sixth, and twelfth month following the index date, respectively (Figure 5).

### Sensitivity analyses

Consistent results were found from the two sensitivity analyses performed on data subsets that (1) did not exclude patients with AS and (2) excluded patients with RA led to consistent findings (results not reported). Among all patients initiated on an oral nonbiologic DMARD, 51% were calculated to have discontinued their index therapy when using a 90-day treatment gap definition for discontinuation, as opposed to the 58% that discontinued in the study analysis with a 60-day treatment gap (results not reported). Additionally, among the entire subgroup of patients initiated on MTX, 44% discontinued their index therapy in the 90-day treatment gap analysis, as opposed to the 52% reported using a 60-day treatment gap. Further, among all patients that were initiated on a biologic DMARD, 36% discontinued their index therapy in the 90-day discontinuation gap analysis, as opposed to the 46% identified using a 60-day treatment gap (results not reported).

## Discussion

This large, retrospective study described nonbiologic and biologic DMARD treatment patterns in patients with PsA based on real-world data gleaned from US-health plans and self-insured employers. Outcome measures included changes documented from the initial DMARD therapy over a 1-year period following treatment initiation, including treatment discontinuations, switches, and therapy add-ons. Recent studies have analyzed treatment patterns for specific biologic DMARDs (adalimumab, etanercept, and infliximab) in patients with RA, psoriasis, PsA, or AS, or in patients with PsA alone [17,18]. Other studies also examined treatment response to different patterns of treatments (for example, investigating the effectiveness of switching to a second TNFi) or the effect of concomitant MTX on treatment response and persistence. However, little is known about treatment patterns when patients are newly initiated on a DMARD, stratified by nonbiologic and biologic DMARDs. This study differs from those reported previously in the literature as it investigates treatment patterns in PsA patients following the initiation of nonbiologic and biologic DMARDs by separately grouping all patients initiated on various nonbiologic DMARDs together in one group, while pooling all patients initiated on various biologic DMARDs together in another study group.

The results revealed that in a sample of 1,698 commercially insured PsA patients newly initiated on an oral nonbiologic DMARD, mainly MTX, patient persistence with treatment was generally low and relatively brief. Almost 70% of these patients had at least one therapy change within the first year, with the initial change generally occurring within the first 3 months after the index date. Moreover, among patients who had at least one therapy change, the majority (83%) discontinued treatment after 3 months. Of those patients who switched treatment or had a therapy add-on, most switched to or added on a biologic DMARD. Similar to the findings in this study, investigators have shown that, in patients with RA, treatment persistence with nonbiologic DMARDs - mainly MTX, chloroquine, and sulfasalazine - erodes rapidly and progressively over time, with persistence rates declining from approximately 70% at one year to 34% at 2 years [19]. In the current analysis, only 31% of the patients persisted uninterruptedly with their initial nonbiologic DMARD therapy after one year, and slightly more than half (56%) persisted uninterruptedly with any nonbiologic or biologic DMARD after one year.

For the patients treated with a biologic DMARD, more than half (54%) persisted with their initial therapy throughout the entire 1-year study period and almost three-quarters (70%) remained on PsA therapy. Of the patients who switched therapy, the vast majority (92%) were treated with another biologic DMARD. These results suggest that patients initiated on a biologic DMARD tend to remain on this form of therapy and do not switch back to nonbiologic DMARDs. In addition, very few patients (7%) had a therapy add-on with a nonbiologic DMARD. Other investigators using retrospective data analyses have also found relatively robust treatment persistence with the use of biologic DMARDs in the treatment of PsA [17,18,20]. In these studies, one-year treatment persistence rates for biologic DMARDs ranged from approximately 50% to 70%, which are consistent with our findings. In one of the studies, relatively high rates of persistence after one year were associated with correspondingly high treatment-response rates [20].

In real-world clinical practice, patients may change therapies for different reasons. Although reasons for the low rate of persistence with the initial DMARD therapy were not available in the database, a potential reason for therapy change may include the lack of effectiveness of the DMARD at controlling patient symptoms and slowing disease progression. Patients might also switch or discontinue treatment due to tolerability or safety issues, or occurrence of adverse events, or discontinue treatment due to disease remission or pregnancy. PsA patients may also switch due to persistent activity of psoriasis rather than PsA Other potential reasons may also include changes in the reimbursement policy of healthcare plan or the involvement of multiple physicians in the management of PsA or other economic reasons. Moreover, many healthcare insurance plans require, for reimbursement purposes, that patients first use at least one to two nonbiologic DMARDs prior to using a biologic DMARD. This may also contribute to the high rates of therapy switch to/add-on of a biologic DMARD among patients initiated on a nonbiologic DMARD. However, further analyses would be warranted to confirm the main reasons for treatment changes in PsA patients.

### Study limitations

Our study was subject to the common limitations of retrospective, observational studies based on healthcare claims data. For instance, the severity of PsA symptoms varied among individuals and could presumably affect a patient’s treatment profile. However, claims databases record diagnostic and procedural codes only and do not reveal disease severity. Moreover, claim data typically report diagnoses/procedures associated with the healthcare services provided for reimbursement purposes and might underestimate patients’ other comorbid conditions (not related to diagnoses received or procedures performed) that are not systematically reported by the physician. For example, in this study, there were only 32% of nonbiologic DMARD patients and 37% of biologic DMARD patients who had a recorded diagnosis for psoriasis. Similarly, only 1.6% of biologic DMARD patients and 2.3% of nonbiologic DMARD patients had a recorded diagnosis for obesity during the baseline period. This suggests an underestimation of the prevalence of these conditions. Further, claims databases do not provide any information regarding the underlying reasons for therapy changes. This study also was limited to a 12-month period following the initiation of a DMARD. Further analyses are warranted to observe treatment patterns over a longer period of time. In addition, the influence of corticosteroid use on treatment patterns was not assessed. Moreover, the study covered the period from 2005 to 2009, an interval when the use of biologic DMARDs for PsA was still relatively new. Patterns of biologic DMARD use may have changed in more recent years. Finally, when performing claims data analyses, it may be difficult to ensure that the selected PsA patients have been accurately diagnosed for PsA, especially as some of the core symptoms of PsA also are known to manifest in AS or RA. The two sensitivity analyses around these topics led to findings consistent with the main analysis.

## Conclusion

This retrospective analysis, based on large and geographically broad US health plan data, provides real-world insight into nonbiologic and biologic DMARD treatment patterns in patients with PsA. The results suggest that the majority of patients treated with nonbiologic or biologic DMARDs change therapy early after treatment initiation, with many switching to a biologic DMARD therapy or, in nonbiologic users, adding on a biologic DMARD. Among the initial biologic DMARD patients who end up switching therapies, the majority do so with another biologic DMARD (as opposed to a non-biologic DMARD). These results suggest that a better understanding of factors related to early DMARD treatment changes in PsA patients are needed to optimize treatment.

## Abbreviations

AAD: American Academy of Dermatology
AS: ankylosing spondylistis
CCI: Charlson comorbidity index
DMARD: disease-modifying antirheumatic drug
MTX: methotrexate
NSAID: nonsteroidal anti-inflamatory drug
PsA: psoriatic arthritis
RA: rheumatoid arthritis. TNFi, tumor necrosis factor inhibitor
